# High Guanine and Cytosine Content Increases mRNA Levels in Mammalian Cells

**DOI:** 10.1371/journal.pbio.0040180

**Published:** 2006-05-23

**Authors:** Grzegorz Kudla, Leszek Lipinski, Fanny Caffin, Aleksandra Helwak, Maciej Zylicz

**Affiliations:** **1**International Institute of Molecular and Cell Biology, Warsaw, Poland; **2**Institute of Biochemistry and Biophysics, Polish Academy of Sciences, Warsaw, Poland; University of BathUnited Kingdom

## Abstract

Mammalian genes are highly heterogeneous with respect to their nucleotide composition, but the functional consequences of this heterogeneity are not clear. In the previous studies, weak positive or negative correlations have been found between the silent-site guanine and cytosine (GC) content and expression of mammalian genes. However, previous studies disregarded differences in the genomic context of genes, which could potentially obscure any correlation between GC content and expression. In the present work, we directly compared the expression of GC-rich and GC-poor genes placed in the context of identical promoters and UTR sequences. We performed transient and stable transfections of mammalian cells with GC-rich and GC-poor versions of Hsp70, green fluorescent protein, and IL2 genes. The GC-rich genes were expressed several-fold to over a 100-fold more efficiently than their GC-poor counterparts. This effect was not due to different translation rates of GC-rich and GC-poor mRNA. On the contrary, the efficient expression of GC-rich genes resulted from their increased steady-state mRNA levels. mRNA degradation rates were not correlated with GC content, suggesting that efficient transcription or mRNA processing is responsible for the high expression of GC-rich genes. We conclude that silent-site GC content correlates with gene expression efficiency in mammalian cells.

## Abstract 2

The effect of nucleotide composition on gene transcription is investigated for Hsp70, GFP, and IL-2, which all show increased expression to correlate with increased GC content at codon position 3.

## Introduction

**Figure 1:**
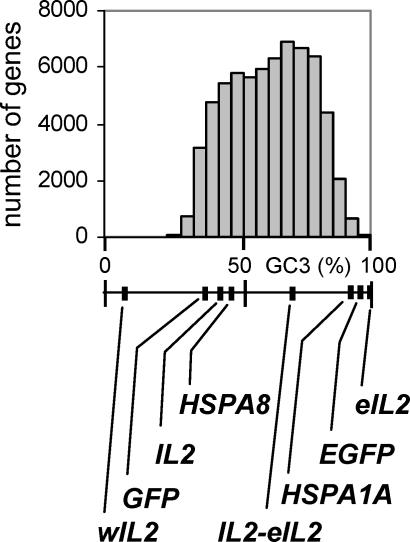
Distribution of Human Genes with Respect to Their GC3 Contents, and GC3 Contents of Genes Used in This Study Data adapted from the Codon Usage Database [ 71].

In the standard genetic code, all U↔C and almost all A↔G substitutions in the third positions of codons are synonymous. Consequently, every protein sequence can be encoded by a large number of different nucleotide sequences, ranging from nearly 0%–100% G and C nucleotides in the third codon positions. In most organisms, the variation in guanine and cytosine (GC) content among genes is modest; for example, 90% of Saccharomyces cerevisiae genes have GC3 contents (proportion of G and C in the third positions of codons) between 30% and 50%. The diversity of codon usage in humans and other mammals is larger than in most other species. The GC3 content of human genes ranges from 20% to more than 95% ( Figure 1). It is believed that this broad variation in nucleotide usage is caused by the large-scale variation of nucleotide composition (isochore structure) of mammalian genomes. Genes located in GC-rich isochores tend to be more GC-rich than genes located in the GC-poor isochores [ 1, 2], and the GC content of pseudogenes increases following their insertion into GC-rich isochores [ 3]. This suggests that the same evolutionary force is responsible for the isochore structure of mammalian genomes and for the codon usage of genes. However, the precise mechanism that underlies the formation of isochores and the diversification of nucleotide usage in genes is not yet clear.

The question of selection on synonymous sites in mammalian genes is widely debated (recently reviewed in [ 4]). In the early studies, silent (synonymous) sites in mammals were assumed to evolve neutrally, and it is still believed that a large majority of silent mutations are neutral. The strongest support for this view comes from an analysis of evolutionary rates at silent sites. Synonymous sites are believed to evolve as fast as the genomic average [ 5], ancient repeats [ 6], and introns [ 7, 8], although some authors report lower silent evolutionary rates [ 9, 10]. Silent substitution rates are also uncorrelated with gene expression breadth and tissue-specificity [ 11]. These results suggest that most synonymous mutations are not opposed by purifying selection in mammals. Furthermore, it is known from studies of bacteria, yeast, and flies that selection intensity on silent sites is correlated with gene expression level, leading to increased codon bias in highly expressed genes in these organisms [ 12– 16]. The lack of clear correlation between codon usage and expression level or breadth in mammals (reviewed in [ 17]) further supports the neutral evolution of silent sites.

And yet, some observations lend support to the existence of selection on silent sites in mammals. The frequency distributions of silent polymorphisms in mammalian genes are compatible with nucleotide usage being determined by selection (or biased gene conversion), but not by regional mutation bias [ 18, 19]. The average GC content is higher at silent sites than in neighboring non-coding regions [ 20], suggesting that high GC content in coding regions could confer some selective advantage. The patterns of tissue-specificity in the codon usage of human genes [ 21, 22], although weak, could indicate translational selection on silent sites. Local codon bias in human genes depends on the position relative to splice sites [ 23, 24], and, as demonstrated in the *CFTR* gene, a surprisingly high proportion of synonymous mutations results in exon skipping and protein inactivation [ 25]. Many human diseases are caused by synonymous mutations resulting in aberrant splicing [ 4]. Finally, synonymous substitution rates vary within mammalian genes, and a case of unusually high sequence conservation at synonymous sites in the *BRCA1* gene has been attributed to selection [ 26]. Most of these arguments are indirect, highlighting the need for experimental studies of mammalian codon usage evolution.

Selection on silent sites requires the existence of functional differences between synonymous genes. Although several cases of such differences have been demonstrated in mammals, they are mostly related to differential splicing of synonymous gene variants. On the other hand, little is known about the effects of nucleotide usage at silent sites on gene expression efficiency. Several recent studies reported weak positive or negative correlations between the GC content and expression levels of mammalian genes [ 17, 27– 32]. All these works relied on estimations of expression levels of endogenous genes using microarrays or analyses of EST or SAGE databases. These are powerful approaches in terms of the amounts of experimental data analyzed. However, since gene expression depends on many factors other than codon usage—such as transcriptional regulation or mRNA UTRs—these studies provide only very indirect insight into the possible effects of codon usage on expression. To eliminate all factors other than nucleotide usage itself, one needs to compare directly the expression of GC-rich and adenine and thymine-rich GC-poor genes, placed in the context of identical promoters and UTR sequences. Here we use this direct experimental approach to study the effects of GC content on the expression of Hsp70 *,* green fluorescent protein (GFP), and IL2 genes in mammalian cells.

## Results

In the first set of experiments, we compared the expression of genes from the mammalian Hsp70 family. We have recently shown that despite the very high similarity of their encoded proteins, mammalian Hsp70-family genes display large differences in their nucleotide usage [ 33, 34]. We used the human *HSPA1A* gene (GC3 = 92%, encoding heat-inducible Hsp70) and the human *HSPA8* gene (GC3 = 46%, encoding constitutive Hsc70). The coding regions of both genes have similar lengths (1,920 and 1,926 nucleotides) and their encoded proteins share 85% identity. To enable a direct comparison of *HSPA1A* and *HSPA8* expression, independent of their genomic context, we cloned their cDNA coding regions into pcDNA3.1 mammalian expression vectors. The 5′- and 3′- UTRs were comprised of the pcDNA3.1 vector sequence, and they were identical in both vectors. HA tags were used to enable easy comparison of protein expression levels, and the first three codons in *HSPA8* were replaced by *HSPA1A* codons to avoid differences in the Kozak translation initiation sequence.

**Figure 2:**
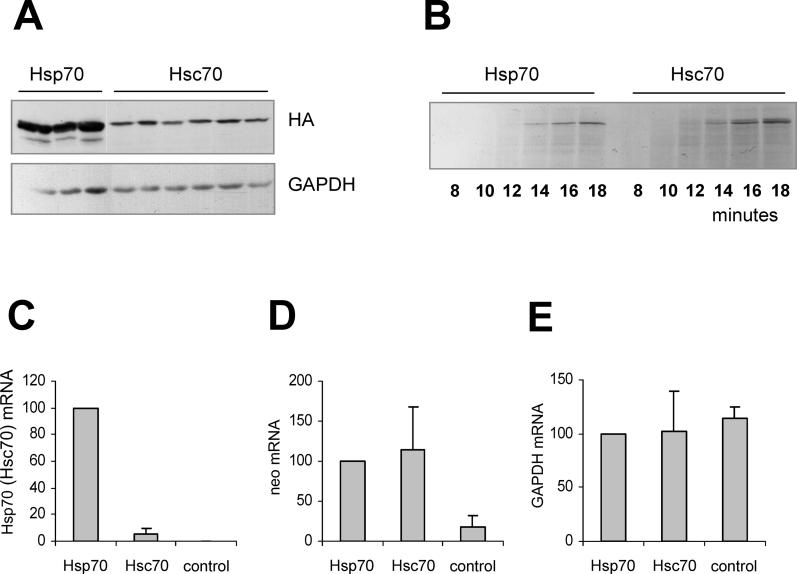
Expression of Hsp70 (GC3 = 92%) and Hsc70 (GC3 = 46%) (A) Three independent clones of pcDNA3-Hsp70-HA (GC3 = 92%) and six clones of pcDNA3-Hsc70-HA (GC3 = 46%) were used to transfect HeLa cells. 24 h following transfection the cells were harvested and the Hsc70-HA or Hsp70-HA protein levels were analyzed by Western blotting using an anti-HA antibody. An anti-GAPDH antibody was used as loading control. (B) Equal amounts of Hsp70 and Hsc70 mRNA were used as templates for in vitro translation in rabbit reticulocyte lysates in the presence of ^35^S-Methionine. The reaction was initiated by the addition of reticulocyte lysate to the translation mix and samples were removed in 2-min intervals into SDS sample buffer. The reaction products were analyzed by SDS-PAGE and autoradiography. (C–E) HeLa cells were transfected with equal amounts of pcDNA3-Hsp70-HA or pcDNA3-Hsc70-HA plasmids. After 24 h, total cellular RNA was isolated and analyzed by qRT-PCR. The graphs represent Hsp or Hsc70 (C), neo (D), and GAPDH (E) mRNA amounts. Hsp70, cells transfected with pcDNA3-Hsp70-HA; Hsc70, cells transfected with pcDNA3-Hsc70-HA; control, untransfected cells. The mRNA amounts were normalized to the amounts in the Hsp70-transfected cells. The error bars represent standard deviations from three to four independent transfections.

We transfected HeLa cells using equal amounts of pcDNA3-Hsp70-HA or pcDNA3-Hsc70-HA vectors. Following 24 h of incubation at 37 °C, the cells were harvested and the Hsp70-HA and Hsc70-HA proteins were quantified by Western blotting using an anti-HA antibody. The Hsp70-HA protein, encoded by the GC-rich gene, was at least ten times more abundant than Hsc70-HA ( Figure 2A). The difference was consistently observed over a 3-fold range of plasmid concentrations ( Figure S1A) and was apparent as soon as 3 h post-transfection, when the Hsp70-HA protein first appeared (unpublished data). Identical results were obtained using 293T cells ( Figure S1B). Since *HSPA1A* is a heat-inducible gene, we tested whether high GC content facilitates its expression at high temperatures. We found that the ratio of Hsp70-HA to Hsc70-HA protein levels did not change with temperature in the range from 28 °C to 42 °C (unpublished data), suggesting that *HSPA1A* expression is enhanced independently of temperature.

Among the 641 codons in the *HSPA1A* gene, 77% are the preferred human codons, i.e., those that are most frequently used in human genes. In comparison, only 39% of the codons in *HSPA8* are preferred human codons. We therefore hypothesized that the difference in Hsp70-HA and Hsc70-HA protein abundance in cells might be due to different translation rates of these proteins. To explore this possibility, we performed in vitro translation experiments. Equal amounts of Hsp70 and Hsc70 mRNA (1.5 μg each) were used for translation in rabbit reticulocyte lysates in the presence of ^35^S-methionine. The only detectable protein products in the translation reactions corresponded to complete Hsp70 and Hsc70 polypeptides. The Hsp70 and Hsc70 proteins both appeared between 12 and 14 min after the reaction started ( Figure 2B). There was no detectible difference in the rates of Hsp70 and Hsc70 translation.

If the translation rates of Hsp70 and Hsc70 are similar, then their different cellular protein levels could arise from a difference in mRNA abundance. To test this possibility, we quantified Hsp70-HA and Hsc70-HA mRNA using real-time RT-PCR, by amplifying a fragment of the 3′ UTR identical in both mRNAs. 24 h after the transfection of HeLa cells, the amount of Hsp70-HA mRNA was over 10-fold higher than the amount of Hsc70-HA mRNA ( Figure 2C). No Hsp70-HA or Hsc70-HA mRNA was detected in untransfected HeLa cells ( Figure 2C). To control for possible differences in transfection efficiencies, we quantified the mRNA of the neomycin resistance *(neo)* gene expressed from both plasmids. The neo mRNA levels were identical in the Hsp70 and Hsc70-transfected cells, suggesting that both plasmids were transfected with equal efficiencies ( Figure 2D). The equal loading of total mRNA in all samples was also confirmed using a cellular housekeeping gene, *GAPDH* ( Figure 2E). Thus, the difference in the Hsp70 and Hsc70 cellular mRNA levels results from their different transcription efficiency or mRNA stability. Similar results were obtained using 293T cells ( Figure S1C–E). Taken together, these results lead to the hypothesis that GC content may strongly affect the expression efficiency of *HSPA1A* and *HSPA8* genes.

**Figure 3:**
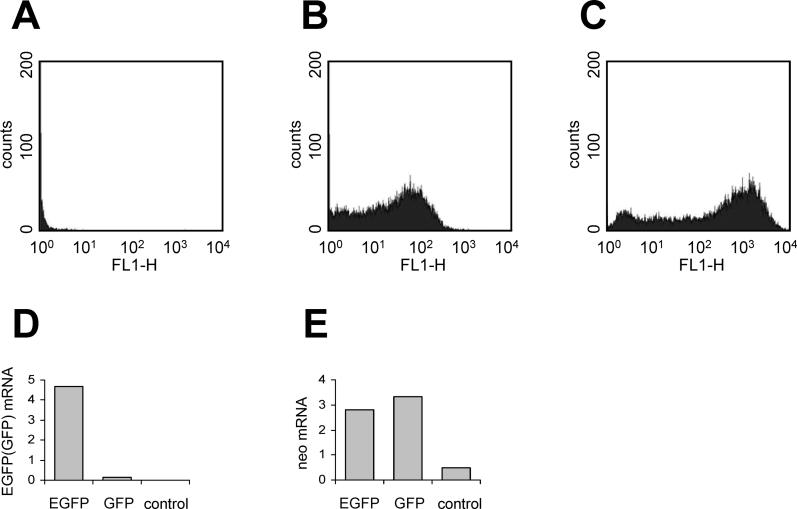
Expression of EGFP (GC3 = 96%) and GFP(GC3 = 35%) in Transiently Transfected Cells (A–C) HeLa cells were transfected with pGFP-N2 or pEGFP-N2 plasmids. 24 h following transfection, cells were trypsinized and washed, and GFP and EGFP protein levels were analyzed by flow cytometry. (A) Control cells. (B) Cells transfected with pGFP-N2. (C) Cells transfected with pEGFP-N2. The horizontal axes represent green fluorescence. (D and E) Expression of GFP and EGFP mRNA. HeLa cells were transfected with pGFP-N2 or pEGFP-N2 plasmids. After 24 h, total cellular RNA was isolated and analyzed by qRT-PCR. The graphs represent GFP or EGFP (D) and neo (E) mRNA amounts. Control, untransfected cells. The results are representative of three experiments.

To test the possibility that high GC content might increase gene expression in mammalian cells, we used plasmids encoding either a modified GC-poor jellyfish *GFP* gene (GC3 = 35%) or a GC-rich version of the gene, *EGFP* (GC3 = 96%). The Kozak sequences of both genes, the encoded protein sequences, and the plasmid sequences around the genes were identical. 24 h following transfection of HeLa cells, the overall EGFP fluorescence was 20–30 times higher than GFP fluorescence ( Figure 3A–C). The same result was seen in 293T cells and at times ranging from 6–36 h post-transfection (unpublished data), in agreement with previous reports [ 35]. We next investigated the amounts of GFP or EGFP mRNA produced in transiently transfected HeLa cells. mRNA was quantified by real-time RT-PCR, using a fragment of the 3′ UTR that was identical in both genes. As shown in Figure 3D, the steady-state level of EGFP mRNA was 20–50 times higher than that of GFP mRNA in HeLa cells. As a control, neo mRNA levels were similar for both plasmids, suggesting that the transfection efficiencies of pGFP-N2 and pEGFP-N2 plasmids did not differ ( Figure 3E). The same results were obtained in 293T cells (unpublished data). Since the ratio of EGFP to GFP mRNA levels was similar to the ratio of their protein levels, it is reasonable to conclude that mRNA level and not translation rate is responsible for the efficient EGFP protein synthesis in human cells.

It is usually believed that selection on silent sites does not significantly affect codon usage in mammals. Thus, even if a gene becomes GC-poor and inefficiently expressed, perhaps because of its location in a GC-poor isochore, selective forces are not strong enough to improve the codon usage of that gene. It follows that many mammalian genes may have codon usage patterns that do not support their efficient expression. We analyzed human genes used in biotechnology or pharmaceutical industry. Several of them have GC3 contents below 60%, the median GC3 content of human genes ( Table S1). To test whether the expression of these genes could be modulated by changing their GC content, we used synthetic nucleotide usage variants of the *IL2* gene.

The IL2 protein is produced by T cells in response to antigenic stimulation. It performs a variety of immunostimulatory functions, including the induction of proliferation of T and B lymphocytes [ 36]. Recombinant IL2 (as Proleukin) is used in therapy of metastatic renal cell carcinoma and metastatic melanoma, and cancer gene therapy trials using IL2 cDNA are ongoing [ 37– 39]. An important factor in gene therapy and biotechnology is the efficiency of therapeutic gene expression. Since the original human *IL2* gene has a low GC content (GC3 = 41%) that could potentially hamper its expression, we attempted to enhance IL2 expression using a synthetic version of the gene, *eIL2* (enhanced *IL2,* GC3 = 100%). To provide additional controls for the relationship between GC content and expression, we used *wIL2* (weakened *IL2,* GC3 = 7%), and a fusion gene containing half of the *IL2* gene and half of the *eIL2* gene ( *IL2-eIL2,* GC3 = 70%). All four IL2 constructs encode exactly the same protein sequence, and they were cloned into the pcDNA3.1 vector using the same restriction sites.

**Figure 4:**
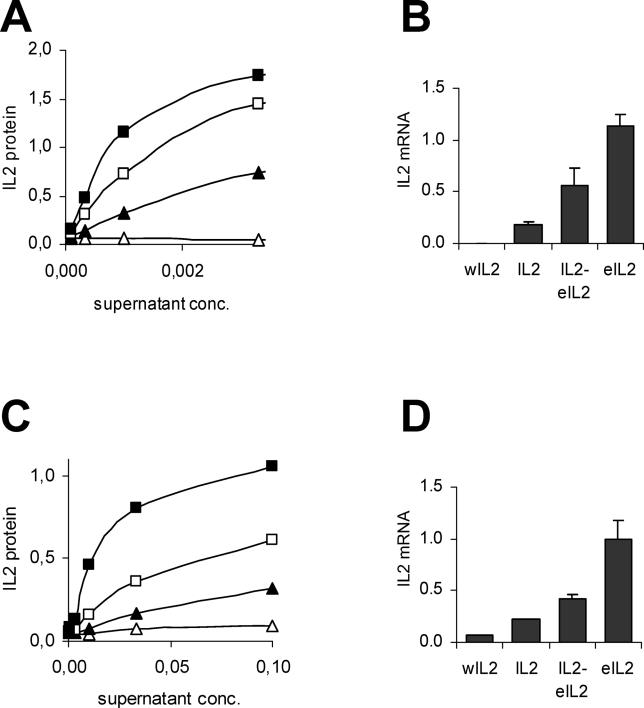
Expression of Human Interleukin 2 (IL2) Variants in Transiently Transfected Cells (A and B) HeLa cells were transfected with plasmids encoding the IL2 variants. 24 h following transfection, cell culture media were used for protein quantification by ELISA, and adhering cells for mRNA measurements by real-time RT-PCR. (A) ELISA measurement of IL2 protein levels using serial dilutions of culture media. Black squares, enhanced IL2 (eIL2, GC3 = 100%); white squares, IL2-eIL2 hybrid (IL2-eIL2, GC3 = 70%); black triangles, wild-type IL2 (IL2, GC3 = 41%); white triangles, weakened IL2 (wIL2, GC3 = 7%). The result is representative of three experiments. (B) Real-time RT-PCR measurement of IL2 mRNA. IL2 mRNA levels were normalized to GAPDH mRNA. Error bars represent standard deviations from two to four independent transfections using different plasmid preparations. (C and D) same as (A and B) using Saos-2 cells.

The production of IL2 protein from the four constructs was measured by ELISA in cell culture supernatants. As expected, IL2 protein synthesis increased with increasing GC content of the genes ( Figure 4A and 4C). In HeLa cells, the *eIL2* gene was expressed 5-fold stronger, and the *IL2-eIL2* hybrid 3-fold stronger than the original *IL2* gene. The expression of the *wIL2* gene was so weak that the protein was not detectable in the HeLa cell culture media. In Saos-2 cells, protein synthesis of *eIL2* and *IL2-eIL2* was 13-fold and 3-fold stronger, respectively, than of wild-type *IL2,* while protein synthesis of *wIL2* was five times lower than wild-type. Real-time RT-PCR experiments demonstrated a very similar positive correlation between GC content and mRNA levels, both in HeLa and Saos-2 cells ( Figure 4B and 4D). These experiments support the hypothesis that the nucleotide usage of mammalian genes can be modified to increase their mRNA levels.

**Figure 5:**
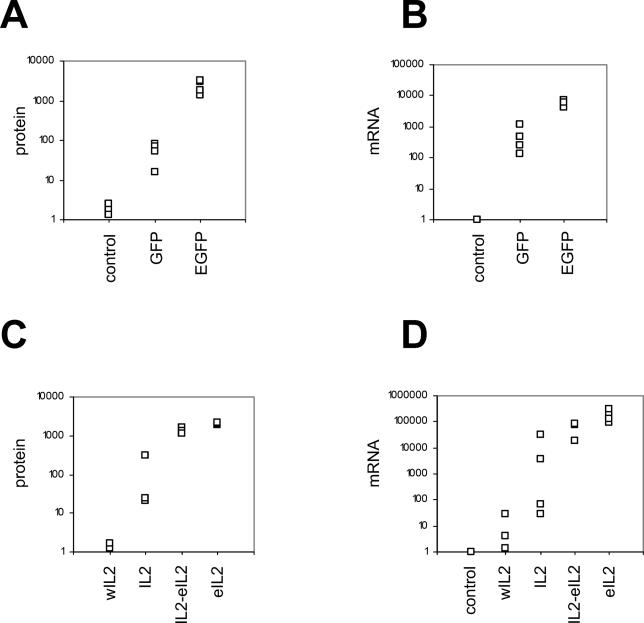
Expression of GFP and IL2 Variants in Stably Transfected MCF-7 Cells MCF-7 cells were stably transfected with expression plasmids containing GFP (GC3 = 35%), EGFP (GC3 = 96%), wIL2 (GC3 = 7%), IL2 (GC3 = 41%), IL2-eIL2 (GC3 = 70%), eIL2 (GC3 = 100%), or with an empty pcDNA3.1 plasmid. The expression plasmids contained CMV promoters and were integrated in random genomic locations. Protein and mRNA was quantified in three to five individual clones for each transgene. (A) Flow cytometry measurements of GFP and EGFP protein levels. (B) Real-time RT-PCR measurements of GFP and EGFP mRNA levels. (C) ELISA measurements of the IL2 protein levels. (D) real-time RT-PCR measurements of IL2 mRNA levels. GFP and IL2 mRNA levels were normalized to GAPDH mRNA. The controls represent pcDNA3.1-transfected cells. The vertical axis in each graph represents arbitrary units.

We next wanted to check whether GC content would affect the expression of genes integrated into mammalian chromosomes, as opposed to genes expressed from plasmids. Stable integration of transgenes eliminates many problems potentially associated with transient transfection, such as unequal plasmid concentration or purity and different transfection efficiency. We first used the MCF-7 human breast cancer cell line to integrate the various GFP and IL2 constructs into random genomic locations. As a negative control, we stably transfected MCF-7 cells with an empty pcDNA3.1 plasmid. Clones were selected using G418, and three to five clones of each type were used for measurements of mRNA and protein levels. As shown in Figure 5A, all the clones containing the GC-rich *EGFP* gene produced 10-fold to 100-fold more fluorescence than the clones expressing the GC-poor *GFP* gene, indicating increased EGFP protein levels. A similar result was obtained when comparing GFP and EGFP mRNA levels ( Figure 5B). The IL2 mRNA and protein levels also correlated very strongly with the GC contents of stably integrated *IL2* gene variants ( Figure 5C and 5D). In this case, the variation in expression levels spanned several orders of magnitude, and considerable variation existed even among clones expressing the same gene (see e.g., Figure 5D, *IL2* gene). However, none of the clones transfected with the GC-poorest *wIL2* gene produced significant amounts of IL2 mRNA or protein ( Figure 5C and 5D).

**Figure 6:**
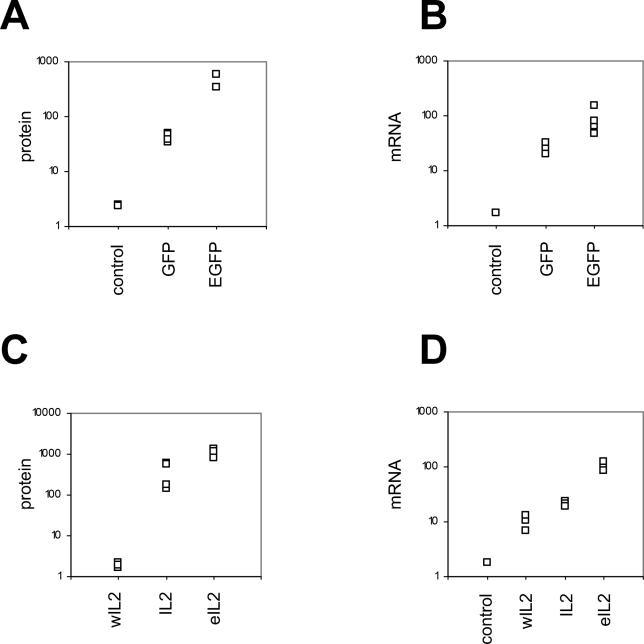
Expression of *GFP* and *IL2* Variants in Stably Transfected Flp-In T-Rex-293 Cells The *GFP* or *IL2* variants under the control of tetracycline-inducible CMV promoters were integrated into the single FRT site of Flp-In T-Rex-293 cells. Protein and mRNA was quantified in three to five individual clones for each transgene. (A) Flow cytometry measurements of GFP and EGFP protein levels 24 h post-induction. (B) Real-time RT-PCR measurements of GFP and EGFP mRNA levels 12 h post-induction. (C) ELISA measurements of the IL2 protein levels 24 h post-induction. (D) Real-time RT-PCR measurements of IL2 mRNA levels 24 h post-induction. GFP and IL2 mRNA levels were normalized to GAPDH mRNA. The controls represent CAT- transfected cells (A) or the parental Flp-In T-Rex-293 cell line (B and D). The vertical axis in each graph represents arbitrary units.

Random genomic integration of transgenes often results in a large variation of expression between clones, due to differences in integration sites or transgene copy numbers. In order to avoid this variation, we next used the Flp-In T-Rex-293 cell line to integrate single copies of the *GFP* or *IL2* variants into a specific genomic location. The Flp-In T-Rex-293 cells also contain a Tet-ON inducible expression system. To eliminate possible artifacts caused by constitutive transgene expression during the selection process, clones were selected in the absence of tetracycline. We then measured the transgene expression following tetracycline addition. After 12–24 h following induction, EGFP mRNA and protein levels were around ten times higher than GFP levels ( Figure 6A and 6B). eIL2-transfected cells produced 5- to 10-fold more transgenic mRNA and protein than IL2-transfected cells ( Figure 6C and 6D). In contrast, the wIL2 protein and mRNA levels barely exceeded background measurements in the parental Flp-In T-Rex-293 cell line. Similar results were also obtained with TM3-FRT cells, with site-directed integration and constitutive, CMV promoter-driven expression of the *GFP* and *IL2* transgenes (unpublished data). As expected, the variation between clones was much lower in the cells with site-directed transgene integration than in the cells with random integration sites ( Figures 5 and 6 and unpublished data).

**Figure 7:**
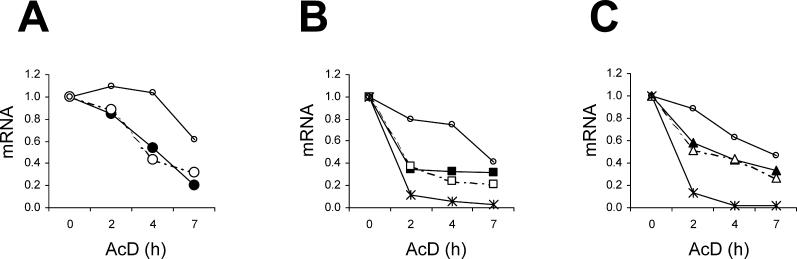
Stability of GC-Rich and GC-Poor mRNA in Mammalian Cells HeLa cells were transfected with the indicated plasmids and after 20 h they were treated with 10 μg/mL actinomycin D. At the indicated times, mRNA was isolated and quantified by real-time RT-PCR. The GAPDH and c-myc mRNA levels represent the means of their levels in cells transfected with GC-rich and GC-poor genes. In each graph, little circles represent GAPDH and crosses, c-myc. (A) black circles, Hsp70; white circles, Hsc70. (B) black squares, EGFP; white squares, GFP. (C) Black triangles, eIL2; white triangles, IL2. The data is representative of two independent experiments.

To test whether slow degradation or efficient synthesis caused the increased steady-state levels of GC-rich mRNA, we performed mRNA stability studies using an inhibitor of transcription, actinomycin D. HeLa cells were transfected with Hsp70, Hsc70, or with the GC-rich or GC-poor versions of *GFP* or *IL2* genes. 20 h following transfection, the cells were treated with actinomycin D for 0–7 h, and mRNA was quantified by real-time RT-PCR. Two cellular mRNA species: GAPDH (stable) and c-myc (unstable) were also quantified to control the proper transcription inhibition by actinomycin D. As expected, the measured half-life of GAPDH mRNA was around 7 h, while the half-life of c-myc mRNA was below 1 h ( Figure 7). The stabilities of GC-rich and GC-poor mRNA species were similar in all cases ( Figure 7). The mRNA half-lives were: Hsp70, 2.9 h; Hsc70, 3.8 h; EGFP, 4.8 h; GFP, 3.3 h; eIL2, 4.5; IL2, 3.9 h. These slight differences in mRNA stabilities lifetimes are unlikely to account for the large difference in steady-state levels of GC-rich and GC-poor mRNA species. This result suggests that enhanced mRNA transcription or co-transcriptional processing accounts for the increased expression of GC-rich genes in mammalian cells.

## Discussion

We have shown that the efficiency of mRNA production from *GFP, IL2,* and Hsp70-family genes in mammalian cells correlates with the silent-site GC content of these genes. Although the origin of GC content variability in human genes attracts much interest, the effect of GC content on gene expression in mammalian cells has not been previously addressed in a direct experimental way. However, previous studies on codon optimization provide some insight into the relationship between nucleotide usage and expression. In mammalian expression systems, the codon optimization strategy consists in increasing the proportion of preferred (i.e., most frequently used) mammalian codons in target genes. Since all of the preferred mammalian codons have G or C nucleotides in the third positions, codon-optimized genes are necessarily GC-rich. We reevaluated the results of published codon optimization experiments by analyzing the effects of GC content on gene expression ( Table 1). All these results support the higher expression of GC-rich genes as compared to adenine and thymine-rich genes. The ratio of GC-rich to adenine and thymine-rich gene expression levels varies from 2.5-fold to over 1,000-fold ( Table 1). This large variation is understandable, considering the different degrees of codon usage modification and different methods for quantifying gene expression. It has often been assumed that the increased expression of codon-optimized genes was caused by a translational mechanism [ 40– 42], although this possibility has not been thoroughly tested experimentally (but see [ 43]). Here we suggest that most of the observed codon optimization effects in mammalian cells may be attributed to expression changes at the mRNA level. For example, optimization of GFP has been assumed to enhance its translation rate [ 40]; instead, we have shown that codon optimization increases GFP mRNA levels.

**Table 1:**
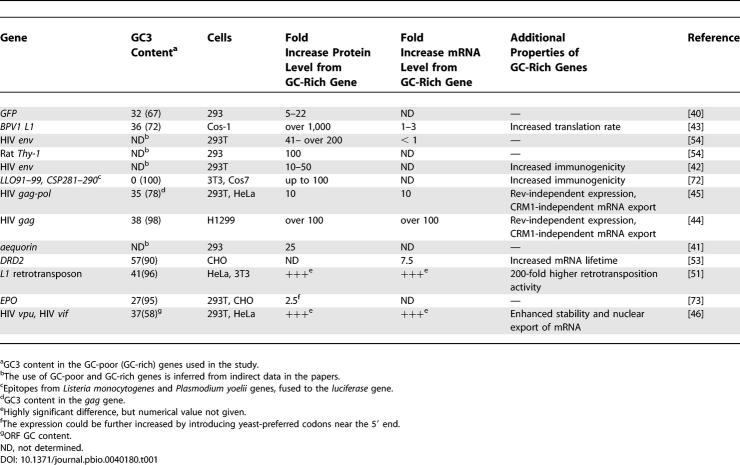
Effects of GC3 Content on Gene Expression in Mammalian Cells

In some of the previous studies, increased mRNA levels contributed to the enhanced protein levels of codon-optimized (GC-rich) genes ( Table 1, [ 44– 46]). A codon-optimized version of HIV-1 *gag* (GC3 = 98%), was expressed in H1299 cells 100-fold more efficiently than wild-type *gag* (GC3 = 38%), both at mRNA and protein levels [ 44]. Unlike the wild-type gene, codon-optimized *gag* was expressed independently of the cis-acting mRNA regulatory elements, and did not require the RNA-interacting protein Rev for efficient expression [ 44, 47]. Similar effects of codon optimization were also shown for the HIV-1 *gag-pol* gene [ 45] and for HIV-1 *vif* and *vpu* genes [ 46]. The latter study demonstrated that codon optimization enhanced nuclear export, but not the transcriptional efficiency of vif and vpu mRNA. Furthermore, the GC-poor HPV-16 *L1* (GC3 = 26%) and *L2* genes (GC3 = 16%) as well as BPV-1 *L1* and *L2* genes (GC3 = 36%) have been shown to contain potent cis-acting mRNA-down-regulating elements in their open reading frames (ORF) [ 48, 49]. The HPV-16 *L2* elements operate in an orientation-dependent manner, and their effect is partially explained by cytoplasmic RNA destabilization [ 48]. Most interestingly, the effects of L1 and L2 inhibitory elements could be overcome by T7 polymerase-driven cytoplasmic transcription in a vaccinia virus-based system, suggesting that most of the mRNA down-regulation takes place at the stage of transcription or nuclear export [ 48, 50]. A recent study of the mammalian GC-poor L1 retrotransposon expression shows that its mRNA is down-regulated at posttranscriptional or transcriptional levels depending on the ORF sense or antisense orientation [ 51, 52]. Increasing the proportion of TpA dinucleotides in the human *DRD2* gene lowered its mRNA stability, while increasing the proportion of CpG dinucleotides increased the stability [ 53]. Finally, two codon optimization studies failed to detect differences in the levels of GC-rich and adenine and uracil (AU)-rich mRNAs [ 43, 54]. In one of these works, different probes were used to compare GC-rich and AU-rich mRNA abundance without correction for hybridization efficiency, weakening the conclusions [ 43]. Taken all together, results obtained in most prior studies are compatible with our hypothesis that high GC content enhances mRNA levels in human cells.

The increased mRNA levels of GC-rich genes detected in this and previous studies can result from two mechanisms, not mutually exclusive: increased mRNA synthesis or decreased mRNA degradation. Interestingly, both RNA synthesis and degradation could potentially be affected by GC content in coding regions. It is well documented that AU-rich elements located in the 3′ UTRs can act to destabilize mRNA [ 55– 57]. cis-acting RNA-destabilizing elements have also been detected in the coding regions of several genes [ 50, 58– 61], but they remain poorly characterized. Most of the mRNAs that harbor coding region instability elements happen to be GC-poor (i.e., factor VIII, IL2, c-myc, c-fos, HPV, and HIV-1 mRNAs), but it is not known whether a general correlation exists between cellular mRNA lifetime and GC3 content. On the other hand, low GC content might also be associated with low transcription or RNA processing efficiency. The efficient expression of GC-poor genes in a T7 polymerase-driven transcription system in mammalian cells supports this type of mechanism. Low RNA-DNA duplex stability [ 62] and runs of uridines [ 63] have been implicated in abnormal pausing and arrest of mammalian RNA polymerase II, and U-rich motifs as well as the conserved AAUAAA signal play a role in normal transcription termination [ 64]. High GC content could also facilitate DNA transitions from B to A or Z conformation [ 65, 66], thereby affecting transcription factor binding or RNA polymerase processivity [ 67, 68]. The possible effects of DNA conformational transitions on nucleotide usage evolution have been previously described [ 69].

To distinguish between the effects of GC content on mRNA synthesis versus mRNA degradation in this study, we performed actinomycin D chase experiments. We demonstrated that GC content does not significantly affect the cellular mRNA lifetimes of *GFP, IL2,* and *Hsp70* genes. Further, we have shown that destruction of the single AU-rich element-like sequence element in the GFP coding region does not enhance GFP expression (unpublished data), suggesting that AU-rich element-mediated RNA destabilization is not responsible for the low GFP expression. Taken together, these results suggest that the high expression of GC-rich genes results primarily from the efficient production of polyadenylated mRNA, through efficient transcription or co-transcriptional processing. The observation that codon usage optimization can enhance gene expression in homologous systems (as in the case of the IL2 optimization) may have important implications for biotechnology and medicine.

It is important to note that we have neither proved nor disproved the idea that selection determines codon usage in mammalian genes. While the differences in expression levels between GC-rich and GC-poor genes are very important, single AT↔GC substitutions may only cause minor changes in expression. The selective coefficients associated with such minor changes may be too small to affect the evolutionary outcomes in small mammalian populations. It might be tempting to hypothesize that the paucity of GC-poor (GC3 < 25%) genes in mammalian genomes is related to their very low expression efficiency. Ultimately, population genetical studies will be required to address these issues. Our results suggest that selection on silent sites is in principle possible in mammals, and that it is more likely to act through transcriptional than translational mechanisms.

Interestingly, the correlation between GC content and mRNA levels has not been detected in most genome-wide microarray and SAGE studies [ 17, 28, 70]. This might suggest that the correlation reported here concerns only a limited number of genes. However, given the wealth of experimental data supporting the increased expression of GC-rich genes (both here and in previous studies on codon optimization), we believe that this phenomenon may be general. Since the GC content of a gene changes slowly, the effects of GC content on expression could be easily compensated by relatively faster changes in promoter, UTR, or intron sequences. Such compensation would decrease the correlation observed in genome-wide studies, which do not control for the genomic context of each gene. A comparative analysis of mammalian and lower vertebrate genes could resolve whether or not GC content changes within genes are associated with compensating changes in their regulatory sequences.

## Materials and Methods

### Plasmids.

pcDNA3.1(+) and pcDNA3.1(−) were from Invitrogen (Invitrogen, Carlsbad, California, United States). These plasmids contain a strong constitutive CMV promoter, a BGH polyadenylation signal, and a neomycin resistance gene for G418-based selection of mammalian cells.

pcDNA3-Hsp70-HA: the *HSPA1A* (gi: 188487) coding region was amplified by PCR using the Hsp70-HA-U and Hsp70-HA-L primers (see Table S2). This appended an HA tag to the Hsp70 ORF. The PCR product was digested with EcoRV and XhoI and inserted into the EcoRV and XhoI sites of the pcDNA3.1 vector (Invitrogen), under the control of the CMV promoter.

pcDNA3-Hsc70-HA: the *HSPA8* (gi: 32466) coding region was amplified by PCR from HeLa cDNA using the Hsc70-HA-U and Hsc70-HA-L primers ( Table S2). This replaced the first three Hsc70 amino acids with Hsp70 amino acids, improved the Hsc70 Kozak sequence, and appended an HA tag to the Hsc70 ORF. The PCR product was digested with EcoRV and XhoI and inserted into the EcoRV and XhoI sites of the pcDNA3.1 vector (Invitrogen).

pEGFP-N2 was from Clontech (Clontech, Palo Alto, California, United States).

pGFP-N2 was constructed by first introducing the R80Q mutation into the GFP sequence in the pS65T-C1 vector (Clontech) using the GFP-R80Q-U and GFP-R80Q-L primers, then by introducing the F64L mutation, using the GFP-F64L-U and GFP-F64L-L primers, and then by amplifying the coding region of the modified GFP using the BamHI-5′-GFP and GFP-3′-NotI primers ( Table S2). The PCR product was digested with BamHI and NotI and then inserted into the BamHI and NotI sites of the pEGFP-N2 vector. The resulting pGFP-N2 vector encoded a GFP with an identical amino acid sequence and Kozak sequence as pEGFP-N2 (see Dataset S1).

pcDNA3-IL2 was constructed by extracting the IL2 cDNA from pWPXL-IL2 (kind gift from D. Kowalczyk) using BamHI and EcoRI and insertion into the BamHI, EcoRI sites of pcDNA3.1 (+).

pcDNA3-eIL2 and pcDNA3-wIL2 were constructed by introducing the synthetic *eIL2* or *wIL2* genes (ordered from Geneart, Regensburg, Germany) into the HindIII, EcoRI sites of pcDNA3.1 (+). The sequences of *eIL2* and *wIL2* can be found online in the Dataset S1.

pcDNA3-IL2-eIL2 was constructed from pcDNA-IL2 by replacing a fragment of the *IL2* gene by a fragment of the *eIL2* gene, PCR-amplified from pcDNA-eIL2 using the eIL2-1152-U and eIL2-1480-L primers ( Table S2) and digested with XbaI.

pcDNA5/FRT-IL2, pcDNA5/FRT-wIL2, and pcDNA5/FRT-eIL2 were constructed by extracting the IL2, wIL2, and eIL2 coding regions, respectively, from pcDNA3-IL2, pcDNA3-wIL2, and pcDNA3-eIL2 using HindIII and NotI and inserting them into the HindIII, NotI sites of pcDNA5/FRT/TO (Invitrogen).

pcDNA5/FRT/CAT was from Invitrogen.

pcDNA5/FRT-GFP and pcDNA5/FRT-EGFP were generated by subcloning the BamHI, NotI fragments from pEGFP-N2 or pGFP-N2 into the pcDNA5/FRT/TO vector digested with BamHI and NotI.

Following cloning, the coding regions of all plasmids were sequenced.

### Cell culture.

Adherent HeLa cells and 293T cells were cultured at 37 °C in a humidified atmosphere containing 5% CO _2_, in Dulbecco's Modified Eagle's Medium (DMEM, Sigma D5523, Sigma, St. Louis, Missouri, United States) with 10% heat-inactivated Fetal Bovine Serum (FBS, Sigma F7524) and the antibiotic/antimycotic mixture (Sigma). Saos-2 cells ( ATCC) were cultured in McCoy's medium with 15% non-inactivated FBS and the antibiotic/antimycotic mixture. MCF-7 cells ( ATCC) were grown in RPMI-1640 (Sigma) containing 10% FBS. For stable transfection of MCF-7 cells, linearized plasmids were transfected using Lipofectamine 2000 (Invitrogen), and clones were selected using 750 μg/mL neomycin (G418 Sigma). The Flp-In T-Rex-293 cell line (Invitrogen) and Flp-In TM3 cells (mouse Leydig cells, L. Lipinski, unpublished data) were cultured in DMEM with 4.5 g/mL glucose, 10% FBS, 100 μg/mL zeocin. 15 μg/mL blasticidin was additionally used for the Flp-In T-Rex-293 cell line. Following the transfection of Flp-In cells, stable transfectants were selected using 100 μg/mL hygromycin B instead of zeocin. Generation of clonal cell lines was performed according to manufacturer's instructions (Flp-In T-Rex Core Kit, Instruction manual, Invitrogen). Total cellular DNA of individual Flp-In T-Rex-293-derived and Flp-In TM3-derived clones was analyzed by qPCR to confirm the presence of a single transgene copy in each clone. GFP and IL2 expression in Flp-In T-Rex-293 cells was induced by adding 1 μg/ml tetracycline for 24 h before harvesting.

For transient transfection of HeLa cells, 5.5 × 10 ^4^ cells per well were seeded in a 24-well plate (Corning, New York, United States). For each well, 0.3 μg plasmid DNA and 1 μL Lipofectamine 2000 (Invitrogen) were used according to the manufacturer's instructions. Following 24 h of incubation, 50%–80% transfection efficiency and > 95% cell viability was routinely achieved, as detected by fluorescence microscopy, immunofluorescence microscopy, and flow cytometry. For transfection of 293T cells, 8 × 10 ^4^ cells per well were used in a 24-well plate. For each well, 0.4 μg pure plasmid DNA was mixed with 25 μL DMEM without FBS, and 0.8 μL 1 mg/mL polyethyleneimine (PEI, Polysciences Incorporated, Warrington, Pennsylvania, United States) in H _2_O was added to this mixture, incubated 10 min at room temperature and the solution was added onto the cells. The transfection efficiency and cell viability was similar as for HeLa cells. For transfection of Saos-2 cells, 1.6 × 10 ^5^ cells per well were seeded in a 12-well plate. For each well, 0.8 μg DNA was mixed with 50 μL DMEM, 1.6 μL of 1 mg/mL PEI was added, incubated 10 min, and spread on the cells. Transfection efficiency was 20%. For mRNA quantification, all transfections were scaled up to 6-well plates.

### SDS-PAGE and Western blotting.

Cells were washed once with ice-cold PBS and lysed directly in the wells in 70 μL 1 × SDS sample buffer, boiled for 5 min and amounts corresponding to about 5 μg total protein per lane were loaded on 10% poliacrylamide gels. A prestained protein ladder (PAGE-Ruler, Fermentas, Burlington, Ontario, Canada) was routinely used. Following electrophoresis, proteins were transferred onto a nitrocellulose membrane (Pall) using a Bio-Rad blotting system (Bio-Rad, Hercules, California, United States). The following antibodies were used for detection: rabbit anti-HA, sc-805 (Santa Cruz Biotechnology), 1:2000; rabbit anti-GAPDH, sc-25778 (Santa Cruz Biotechnology), 1:6000; goat anti-rabbit IgG-HRP conjugated, 401393 (Calbiochem, San Diego, California, United States), 1:6000. The membranes were soaked in the chemiluminescence reagent immediately before exposure to a Kodak BioMax film.

### Flow cytometry.

Cells were trypsinized, washed with medium containing 10% FBS, resuspended in PBS with 5% DMSO, and stored at −70 °C. The flow cytometry analysis was performed using BD FACS Calibur. Forward scatter and side scatter measurements were used to define a homogenous population of living cells, and the FL1 channel was used to detect the GFP or EGFP fluorescence. For fluorescence quantification, the arithmetic mean of all events corresponding to living cells was used.

### IL2 ELISA.

24 h following transfection, cell culture media were gathered and centrifuged 1 min at 14,000 rpm. Supernatants were diluted to the appropriate concentration with PBS + 10% heat-inactivated FBS, and IL2 concentrations were measured using the OptEIA human IL2 ELISA set (BD Biosciences, Palo Alto, California, United States) according to the manufacturer's instructions.

### In vitro transcription and translation.

Capped Hsp70 and Hsc70 mRNA was produced in vitro using the T7 Cap Scribe kit (Roche, Basel, Switzerland) according to the manufacturer's instructions. The mRNA was analyzed by 1% agarose gel electrophoresis to confirm the absence of degradation. The in vitro translations were performed at 28 °C using the Reticulocyte Translation Kit Type II (Roche) and ^35^S-labeled Methionine (Amersham Biosciences, Little Chalfont, United Kingdom). The reactions contained 1–2 μg Hsp70 or Hsc70 mRNA, 2 μL translation reaction mix without methionine, 50 mM potassium acetate, 1.25 mM magnesium acetate, 2 μL ^35^S-Met (10 mCi/mL), and 10 μL rabbit reticulocyte lysate, in a total reaction volume of 25 μL. The reactions were started by the addition of rabbit reticulocyte lysate, and stopped after the desired time by addition of SDS sample buffer, followed by SDS-PAGE and autoradiography.

### mRNA quantification.

Total cellular RNA was purified using the NucleoSpin kit (Macherey Nagel, Germany) according to the manufacturer's instructions. The NucleoSpin purification procedure comprises on-column DNA digestion using DNAse I. On several occasions, we verified the absence of contaminating plasmid DNA in our RNA preparations by omitting the reverse transcriptase in the RT reactions and then performing the real-time PCR. We never observed any significant contamination with this purification method. RNA concentration was measured spectrophotometrically, and approximately 1.5 μg of total RNA was used in each cDNA synthesis reaction. cDNA synthesis was performed using the RevertAid kit (Fermentas) with (dT) _18_ primers. Real-time PCR cDNA quantification was performed using Light-Cycler (Roche) with Sybr Green II (Sigma). The primer sequences are shown in the Table S2. The equal transfection efficiency in transient transfection experiments was controlled using the neomycin resistance gene (neo), present in all our experimental constructs. The neo gene cDNA from the pEGFP-N2 and pGFP-N2 plasmids was amplified using the neo(GFP) primers, and the neo gene cDNA from the pcDNA3-Hsp70-HA, pcDNA3-Hsc70-HA, and all the pcDNA3-IL2 plasmids—using the neo(pcDNA) primers. The IL2 and GFP variants expressed in the Flp-In cells were quantified using the pcDNA5-UTR-U and pcDNA5-UTR-L primers. For RNA stability assays, cells were treated with 10 μg/mL actinomycin D (Sigma) for 0–7 h before RNA isolation. mRNA half-lives were determined by fitting exponential decay curves to experimental data points.

## Supporting Information

Dataset S1 Sequences of the *IL2* and *GFP* Gene Variants
(3 KB TXT)

Figure S1 Expression of Hsp70 (GC3 = 92%) and Hsc70 (GC3 = 46%) in HeLa and 293T Cells (A) HeLa cells were transfected using 0.1, 0.2, or 0.3 μg of pcDNA3-Hsp70-HA or pcDNA3-Hsc70-HA plasmids and the protein expression levels 24 h after transfection were analyzed by Western blotting. (B) Same as (A), using 293T cells. (C–E) 293T cells were transfected with equal amounts of pcDNA3-Hsp70-HA or pcDNA3-Hsc70-HA plasmids. After 24 h, total cellular RNA was isolated and analyzed by qRT-PCR. The graphs represent Hsp/c70 (C), neo (D), and GAPDH (E) mRNA amounts. Hsp70, cells transfected with pcDNA3-Hsp70-HA; Hsc70 cells, transfected with pcDNA3-Hsc70-HA; control, untransfected cells. The mRNA amounts were normalized to the amounts in the Hsp70-transfected cells. The error bars represent standard deviations from 3–4 independent transfections.
(71 KB PDF)

Table S1 Human GC-Poor Genes (GC3 < 60%) Used in Therapy (35 KB DOC)

Table S2 Primers Used for Cloning and Real-Time RT-PCR (44 KB DOC)

## Abbreviations

AU: adenine and uracil
GC: guanine and cytosine
GFP: green fluorescent protein
neo: neomycin resistance
ORF: open reading frame
