# Supplementary material for: High Guanine and Cytosine Content Increases mRNA Levels in Mammalian Cells
Source: PLoS Biol. 2006 May 23;4(6):e180. doi: 10.1371/journal.pbio.0040180 (PMC1463026; doi:10.1371/journal.pbio.0040180)
Supplement: Supplementary file 1 [file pbio.0040180.sg001.pdf]

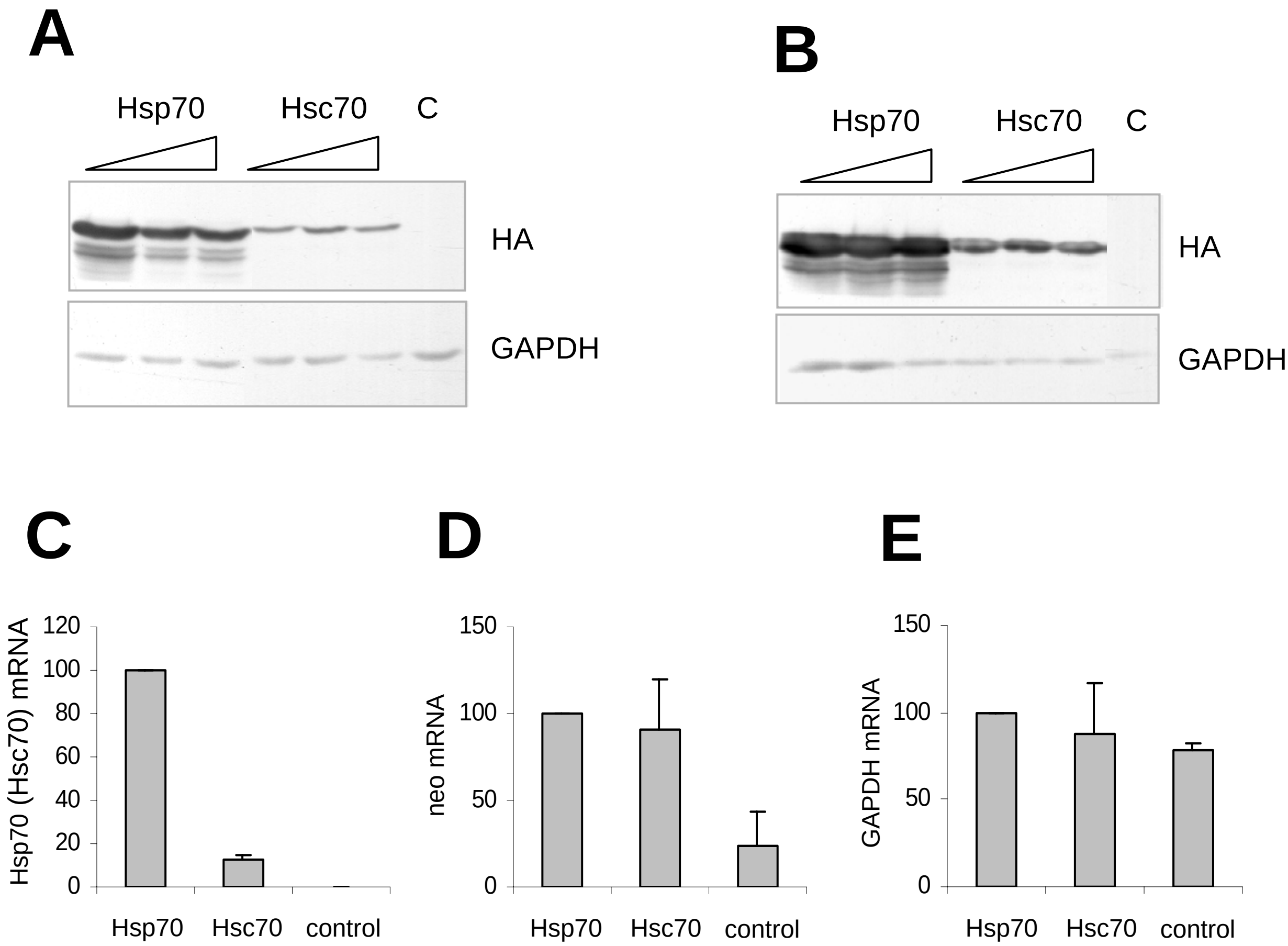
A
Hsp70
Hsc70
C
HA
GAPDH
B
Hsp70
Hsc70
C
HA
GAPDH
C
Hsp70 (Hsc70) mRNA
0
20
40
60
80
100
120
Hsp70
Hsc70
control
D
neo mRNA
0
50
100
150
Hsp70
Hsc70
control
E
GAPDH mRNA
0
50
100
150
Hsp70
Hsc70
control

Figure S1
