# Supplementary material for: High Guanine and Cytosine Content Increases mRNA Levels in Mammalian Cells
Source: PLoS Biol. 2006 May 23;4(6):e180. doi: 10.1371/journal.pbio.0040180 (PMC1463026; doi:10.1371/journal.pbio.0040180)
Supplement: Supplementary file 2 [file pbio.0040180.st001.doc]

Table S1. Human GC-poor genes (GC3<60%) used in therapy.

| Gene | Expasy accession number | GC3 (%) | Protein Length (aa) | Drug, Disease |
| --- | --- | --- | --- | --- |
| Lamina-associated polypeptide 2 isoform alpha | P42166 | 40 | 694 | Timunox (Cilag), Sintomodulina (Italofarmaco) and Mepentil (Recordati). Immune deficiencies, autoimmunity, cancer |
| Interleukin 2 | P60568 | 41 | 153 | Proleukin, Aldesleukin. Metastatic melanoma, renal carcinoma |
| Coagulation factor VIII | P00451 | 41 | 2351 | Haemophilia |
| Thyrotropin alpha, beta chain | P01222 | 42 | 138 | Thyrogen (Genzyme). |
| Interferon gamma | P01579 | 44 | 166 | Actimmune (Genentech). |
| gonadoliberin I | P01148 | 48 | 92 | Factrel (Ayerst Labs) Relisorm (Serono). |
| Alpha-galactosidase A | P06280 | 51 | 429 | Fabry syndrome |
| Thyrotrophin alpha, alpha chain (Choriogonadotropin alpha chain) | P01215 | 55 | 116 | Thyrogen (Genzyme). |
| Ciliary neurotrophic factor | P26441 | 55 | 200 | Axokine, regeneron. Motor neuron diseases |
| Interleukin 12 alpha | P29459 | 55 | 220 |  |
| Follitropin beta chain | P01225 | 58 | 129 | Gonal-F or Metrodin HP (Serono) and Puregon. |
| Adiponectin | Q15848 | 58 | 244 | Diabetes type 2 |
| Arylsulfatase B |  | 59 | 533 | Mucopolysaccharidosis VI |
| Interleukin 6 | P05231 | 59 | 213 | Gene therapy |
