# Supplementary material for: High Guanine and Cytosine Content Increases mRNA Levels in Mammalian Cells
Source: PLoS Biol. 2006 May 23;4(6):e180. doi: 10.1371/journal.pbio.0040180 (PMC1463026; doi:10.1371/journal.pbio.0040180)
Supplement: Supplementary file 3 [file pbio.0040180.st002.doc]

**Table S2. Primers used for cloning and real-time RT-PCR.**

| name | purpose | Sequence |
| --- | --- | --- |
| Hsp70-HA-U | cloning | CGCCTCGAGCCACCATGGCCAAAGCCGCGGCGATCG |
| Hsp70-HA-L | cloning | CGCGATATCCTAAGCGTAGTCTGGGACGTCGTATGGGTAATCTACCTCCTCAATGGTGG |
| Hsc70-HA-U | cloning | CGCCTCGAGCCACCATGGCCAAGGGACCTGCAGTTGGTA |
| Hsc70-HA-L | cloning | CGCGATATCCTAAGCGTAGTCTGGGACGTCGTATGGGTAATCAACCTCTTCAATGGTGGG |
| GFP-R80Q-U | cloning | GATACCCAGATCACATGAAACAGCATGACTTTTTCAAG |
| GFP-R80Q-L | cloning | CTTGAAAAAGTCATGCTGTTTCATGTGATCTGGGTATC |
| GFP-F64L-U | cloning | CTTGTCACTACTCTGACATATGGTGTTCAATGC |
| GFP-F64L-L | cloning | GCATTGAACACCATATGTCAGAGTAGTGACAAG |
| BamHI-5’-GFP | cloning | ATACGGATCCACCGGCCGGTCGCCACCATGGTGAGTAAAGGAGAAGAACTTTTCAC |
| GFP-3’-NotI | cloning | AGATGCGGCCGCTTTACTTGTATAGTTCATCCATCCCAAGTGTAATCCCAGCAG |
| eIL2_1152_U | cloning | AGTGTCTAGAGGAGGAGCTGAAGCC |
| eIL2_1480_L | cloning | GATCAGCGGAGCTCTAGCA |
| GFP-UTR_U | RT-PCR | CAAGTAAAGCGGCCGCGACT |
| GFP-UTR_L | RT-PCR | GGGGAGGTGTGGGAGGTTTT |
| 70-UTR_U | RT-PCR | TACCCATACGACGTCCCAG |
| 70-UTR_L (BGH reverse) | RT-PCR | TAGAAGGCACAGTCGAGG |
| IL2-UTR_U | RT-PCR | CTCGAGCATGCATCTAGAG |
| IL2-UTR_ L (BGH reverse) | RT-PCR | TAGAAGGCACAGTCGAGG |
| pcDNA5-UTR_U | RT-PCR | CTCAGACAATGCGATGCAAT |
| pcDNA5-UTR_L | RT-PCR | GTTGCCAGCCATCTGTTGTT |
| neo(GFP)_U | RT-PCR | GTGCCCTGAATGAACTGCAA |
| neo (GFP)_L | RT-PCR | TACTTTCTCGGCAGGAGCAA |
| neo (pcDNA)_U | RT-PCR | TCCGGTGCCCTGAATGAACT |
| neo (pcDNA)_L | RT-PCR | TACTTTCTCGGCAGGAGCAA |
| GAPDH_U | RT-PCR | GAAGGTGAAGGTCGGAGTCA |
| GAPDH_L | RT-PCR | GAAGATGGTGATGGGATTTC |
| c-myc_U | RT-PCR | CTCCTACGTTGCGGTCACAC |
| c-myc_L | RT-PCR | CCGGGTCGCAGATGAAACTC |
